# An Extension of the Procedural Deficit Hypothesis from Developmental Language Disorders to Mathematical Disability

**DOI:** 10.3389/fpsyg.2016.01318

**Published:** 2016-09-15

**Authors:** Tanya M. Evans, Michael T. Ullman

**Affiliations:** ^1^Department of Psychiatry and Behavioral Sciences, Stanford University School of MedicineStanford, CA, USA; ^2^Brain and Language Laboratory, Department of Neuroscience, Georgetown UniversityWashington, DC, USA

**Keywords:** procedural deficit hypothesis, math disability, dyscalculia, math, dyslexia, specific language impairment, procedural memory, intraparietal sulcus

## Abstract

Mathematical disability (MD) is a neurodevelopmental disorder affecting math abilities. Here, we propose a new explanatory account of MD, the procedural deficit hypothesis (PDH), which may further our understanding of the disorder. According to the PDH of MD, abnormalities of brain structures subserving the procedural memory system can lead to difficulties with math skills learned in this system, as well as problems with other functions that depend on these brain structures. This brain-based account is motivated in part by the high comorbidity between MD and language disorders such as dyslexia that may be explained by the PDH, and in part by the likelihood that learning automatized math skills should depend on procedural memory. Here, we first lay out the PDH of MD, and present specific predictions. We then examine the existing literature for each prediction, while pointing out weaknesses and gaps to be addressed by future research. Although we do not claim that the PDH is likely to fully explain MD, we do suggest that the hypothesis could have substantial explanatory power, and that it provides a useful theoretical framework that may advance our understanding of the disorder.

## Introduction

Children show marked individual differences in their mathematical abilities (Geary, 1994). Mathematical disability (MD), which includes developmental dyscalculia, is a neurodevelopmental disorder in which math abilities are lower than expected given the individual’s age, where the difficulties are not better accounted for by intellectual disability, other developmental disorders, or neurological or motor disorders (American Psychiatric Association, 2013). MD affects 7–10% of school-age children worldwide (Gross-Tsur et al., 1996; Shalev et al., 2000), and can persist as functional innumeracy into adolescence and adulthood (Geary et al., 2013). Whereas the development of math skills in typically developing (TD) children is characterized by improvements in math performance and more efficient problem-solving strategies (Butterworth, 2005), children with MD continue to rely on immature strategies, and make more calculation errors than their TD peers (Geary et al., 1992).

Mathematical disability is highly comorbid with dyslexia (Lewis et al., 1994; Wilson et al., 2015), and may be comorbid with specific language impairment (SLI) as well (Fazio, 1999; Donlan, 2003; Archibald et al., 2013). It has been suggested that the neurobiological basis for this overlap between MD and dyslexia could be either (1) Additive (from independent neural insults) or (2) Domain general (due to the dysfunction of mechanisms that underlie both domains, in particular of either verbal or non-verbal mechanisms) (Ashkenazi et al., 2013a). Here, we propose a domain-general framework whereby aberrations of procedural memory circuitry may provide explanatory value for these cross-domain impairments.

Previous research suggests that certain neurodevelopmental disorders, in particular those affecting reading and language, may be at least partly explained by the procedural deficit hypothesis (PDH; Ullman, 2004; Ullman and Pierpont, 2005; Nicolson and Fawcett, 2007; Lum et al., 2013, 2014; Ullman et al., accepted). Under this view, dyslexia and SLI may be partly or even largely accounted for by abnormalities of brain structures underlying procedural memory, a system that is critical for learning automatized skills (see the section “The Procedural Deficit Hypothesis of Mathematical Disability” for more on the system). These abnormalities are posited to help explain the observed reading and language difficulties, as well as accompanying impairments of other functions that depend on these brain structures (Ullman, 2004; Ullman and Pierpont, 2005). For example, according to the PDH of SLI, the frontal/basal-ganglia abnormalities in the disorder can explain the observed deficits of procedural memory (e.g., of sequence learning), grammar (which appears to rely on procedural memory; Ullman, 2004, 2016), and other functions (e.g., working memory) that depend on these brain structures (Ullman and Pierpont, 2005; Lum et al., 2014; Ullman et al., accepted).

Here, we propose that this brain-based framework may also apply to MD. The extension of the PDH to MD is primarily motivated by the following factors. First, since MD is comorbid with dyslexia and possibly SLI, these disorders may share causal mechanisms. Second, it seems likely that procedural memory underlies certain aspects of math, particularly automatized math skills, which should thus show deficits following aberrations to this system. Third and more generally, an explanatory account involving learning processes seems reasonable, since math (like reading and language) has to be largely if not entirely learned; moreover, learning difficulties might be expected in a developmental ‘learning disorder’ (American Psychiatric Association, 2013). Finally, like developmental disorders of reading and language, MD is neurodevelopmental in origin, and thus a brain-based account could have substantial explanatory power (Ullman and Pierpont, 2005).

We therefore posit that, like dyslexia and SLI, MD can be at least partly explained by abnormalities of brain structures underlying the procedural memory system – though we emphasize that we do not suggest that all aspects of MD are explained by this hypothesis. Importantly, the PDH of MD makes quite specific predictions, and thus the hypothesis can be directly tested. In this paper, we first provide an overview of the PDH of MD and lay out its main predictions. Next, for each prediction, we briefly examine existing evidence and empirical gaps. Since this is a new hypothesis, little evidence exists thus far. Thus, the goal of this paper is primarily to guide future research to examine the validity and utility of this novel perspective.

## The Procedural Deficit Hypothesis of Mathematical Disability

The PDH posits that MD is at least partly explained by abnormalities of brain structures underlying procedural memory. According to the PDH, these abnormalities, which may be caused by a variety of etiologies, should result in problems with various functions that depend on the affected structures, including procedural memory itself (Ullman and Pierpont, 2005; Ullman et al., accepted).

Procedural memory is relatively well understood, from both animal and human studies (for more details on the system and its functions, including the development of automaticity, see Ullman, 2004, 2016; Doyon et al., 2009; Ashby et al., 2010). (Note the term “procedural” is generally used differently in the math literature, where “procedure” is often used interchangeably with “strategy”; also see the section “Difficulties with Aspects of Math that Depend on Procedural Memory”). The procedural memory brain system underlies the implicit learning and processing of a wide range of perceptual-motor and cognitive skills across domains, including motor skills, navigation, sequences, rules, and categories. (Here, procedural memory refers to a particular brain system, rather than implicit memory more generally, which is how some researchers use the term). This system may be specialized for learning to predict, such as the next item in a sequence or the output of a rule. Learning in procedural memory requires practice, and thus typically takes time. However, what is eventually learned seems to be processed rapidly and automatically. The process of automatization is still not well understood. However, typically an initial stage of rapid improvement in performance is followed by a gradual decrease in the learning rate and a trend toward an asymptote, together with the emergence of automaticity (Korman et al., 2003; Hauptmann et al., 2005).

Procedural memory depends on a network of interconnected frontal, parietal, basal ganglia, cerebellar, and other brain structures (Ullman, 2004, 2016; Doyon et al., 2009; Ashby et al., 2010). Each structure contributes somewhat different functions. For example, the basal ganglia (especially the caudate nucleus) seem to play a critical role in learning and consolidating new skills, particular during early stages, whereas neocortical regions, including frontal areas [especially (pre)motor and related cortex, including BA 6 and BA 44], may be more important for processing skills after they have been automatized. Parietal cortex (especially the intraparietal sulcus and adjacent supramarginal gyrus) also plays a role (Ullman, 2004), perhaps in part as a repository of stored skills (Heilman et al., 1997). Indeed, parietal cortex, including the intraparietal sulcus, seems to play a role in automatization, for both math (Grabner et al., 2009, 2013) and motor skills (Sakai et al., 1998; Hikosaka et al., 2002).

The PDH of MD makes a number of predictions. Here we lay out the five main ones. First, MD should be associated with abnormalities of brain structures underlying procedural memory. Because this is a neuroanatomical hypothesis, it makes no claims as to what etiologies or types of neuropathology should lead to these abnormalities. Indeed, at least in SLI, numerous genetic and environmental factors appear to lead to the basal ganglia abnormalities that may underlie the disorder (Ullman and Pierpont, 2005; Ullman et al., accepted). In principle, any of the brain structures subserving procedural memory could be affected in MD. Thus, the PDH focuses on brain networks, or circuitry, rather than on a specific structure (Ullman and Pierpont, 2005). Given that the various brain structures have different functions, the types of procedural memory dysfunctions in MD should depend on which structure(s) are affected (Ullman and Pierpont, 2005). For example, basal ganglia abnormalities should lead to different types of procedural memory dysfunction than parietal abnormalities. However, MD explained by the PDH is only likely if the abnormalities affect those portions of the structures that actually underlie procedural memory. For example, not all parts of parietal cortex or the basal ganglia play roles in procedural memory, and thus abnormalities of these structures will not necessarily lead to procedural memory deficits (Ullman and Pierpont, 2005). Although it remains to be seen which portions are critical for procedural memory, some patterns are already emerging (e.g., within the basal ganglia, the caudate nucleus seems crucial; see above).

Second, abnormalities of neural substrates that subserve procedural memory could of course lead to dysfunctions of procedural memory itself, such as in the automatization of skills that rely on this system (Ullman and Pierpont, 2005). Such abnormalities may therefore cause impairments of math skills that depend on procedural memory, including their automatization. Since the different brain structures of procedural memory have different functional roles, the nature of the math impairments should depend on which brain structures are affected.

Third, these abnormalities may also be expected to lead to broader impairments of procedural memory, beyond math skills. Even if procedural memory circuits turn out to be subspecialized for different types of procedures, such as for math or grammar (for which there is no clear evidence at this point; Ullman et al., 2014), neurobiological abnormalities seem unlikely to be restricted to this subcircuitry alone, leading to a probability of at least somewhat broader problems with procedural memory (Ullman and Pierpont, 2005). Of course if no such subspecialization for math or certain math skills exists, abnormalities of procedural memory circuitry should also result in broader procedural memory impairments. Thus, MD individuals whose math difficulties are at least partly explained by the PDH may show impairments of other skills that seem to depend on this system, such as perceptual-motor skills, navigation, sequences, rules, categories, grammar, and reading.

Fourth, the posited neurobiological abnormalities may affect non-procedural functions as well, since the abnormalities may also extend beyond portions of the circuitry that subserve procedural memory (Ullman and Pierpont, 2005). For example, the frontal/basal ganglia structures affected in SLI also subserve non-procedural functions such as working memory and temporal processing, which may explain the deficits of these functions in the disorder (Ullman and Pierpont, 2005). Thus, individuals with MD may also have difficulties with apparently non-procedural functions such as working memory, attention, inhibitory control, and temporal processing, all of which depend on brain structures underlying procedural memory (Ullman, 2004; Ullman and Pierpont, 2005).

Fifth, the posited impairments of procedural and non-procedural functions such as of grammar, reading, motor skills, and attention could result in comorbidities between MD and disorders of these domains, such as SLI, dyslexia, developmental coordination disorder (DCD), and ADHD, at least where these disorders are due to abnormalities of brain structures that underlie procedural memory (Ullman, 2004; Ullman and Pierpont, 2005). The presence of particular comorbidities may be explained in part by the particular procedural and non-procedural (sub)circuits that are affected.

To avoid confusion about the nature of the PDH, we emphasize that while mathematical difficulties are predicted to result from procedural memory deficits, they can also be caused by other factors. These could arise either from abnormalities of brain structures that underlie other functions in addition to procedural memory, or from abnormalities of other (completely non-procedural) brain structures (since etiologies that affect procedural memory brain structures could also affect other structures; Ullman and Pierpont, 2005). In either case, non-procedural impairments could lead to math difficulties in various ways. For example, it has been suggested that MD may be explained by impairments of working memory, attention, or inhibitory control (see next paragraph), all of which may result from the neural abnormalities posited by the PDH. Additionally, since verbal abilities may be important for aspects of math (Dehaene and Cohen, 1995; Prado et al., 2011; Evans et al., 2014), any language deficits from abnormalities to non-procedural (or procedural) circuitry could also lead to mathematical difficulties. In sum, the posited existence of individuals whose math difficulties are explained by procedural impairments in no way precludes math deficits explained by non-procedural functions, even in the same, let alone other, individuals.

We summarize the five main predictions of the PDH of MD in **Table 1**, where they are compared with analogous predictions from other accounts of MD (Szucs et al., 2013), in particular the magnitude representation (core numerosity) deficit hypothesis (Piazza et al., 2007, 2010; Rousselle and Noël, 2007; Butterworth, 2010), the spatial working memory deficit hypothesis (Geary, 2004; Rotzer et al., 2009), the attention deficit hypothesis (Ashkenazi et al., 2009; Ashkenazi and Henik, 2010; Hannula et al., 2010; Henik et al., 2011), and the inhibitory control deficit hypothesis (Espy et al., 2004). As can be seen in the Table, although some of the predictions of the PDH are also made by other accounts, the full set of predictions allows them to be distinguished. Moreover, we underscore that whereas most other accounts explain MD largely in terms of processing deficits related to particular functions, the PDH posits the dysfunction of a *brain system*, which is moreover involved in *learning*. Thus, while each competing account can explain a particular non-mathematical deficit (e.g., the working memory deficit hypothesis can account for working memory problems, and resulting math difficulties), as we have seen above the PDH can explain a wide range of deficits, since it is a brain-based rather than functional account. Note that even the magnitude representation deficit hypothesis, which is also neuroanatomically grounded (see **Table 1**), differs in spirit from the PDH, in that it focuses on a single brain structure and a single function, rather than the system-wide approach taken by the PDH, which moreover specifically makes the broader claim that any other functions that depend on these brain structures should also be impaired. Finally, given that math must be learned, and MD is a developmental disorder, moreover one that is characterized as a ‘learning disorder’ (American Psychiatric Association, 2013), a learning account may prove to have important explanatory power.

**Table 1:** Predictions of the procedural deficit hypothesis (PDH) compared to other accounts of mathematical disability (MD).

	Procedural deficit hypothesis	Magnitude representation deficit hypothesis	Spatial working memory deficit hypothesis	Attention deficit hypothesis	Inhibitory control deficit hypothesis
*Prediction 1: Abnormalities of brain structures underlying procedural memory*	Yes	Yes (intraparietal sulcus)	Yes? (not clearly specified)	Yes? (not clearly specified)	Yes? (not clearly specified)

*Prediction 2: Difficulties with aspects of math that depend on procedural memory*	Yes	None of these four hypotheses specifically predict difficulties with those aspects of math posited to depend on procedural memory.			

*Prediction 3: Difficulties with procedural memory in other domains*	Yes	None of these four hypotheses predict difficulties with procedural memory in other domains.			

*Prediction 4: Difficulties with non-procedural functions that rely on brain structures subserving procedural memory*	Yes	Yes (magnitude representation)	Yes (spatial working memory)	Yes (attention)	Yes (inhibitory control)

*Prediction 5: Comorbidity with other developmental disorders that may be explained by the PDH*	Yes	No	No	Possibly ADHD	Possibly ADHD

## Evidence, Gaps, and Future Research

Here, we present evidence to date for each of the five main predictions of the PDH of MD, and identify gaps and future areas of research.

### Abnormalities of Brain Structures Underlying Procedural Memory

Math disability explained by the PDH should be accompanied by abnormalities in one or more brain structures that underlie procedural memory. A number of these brain structures have already been implicated in MD, even though these abnormalities have thus far not been interpreted from the perspective of the PDH.

Perhaps the most consistently implicated procedural memory brain structure in MD to date is parietal cortex, in particular the intraparietal sulcus, with both structural (Molko et al., 2003; Rotzer et al., 2008; Rykhlevskaia et al., 2009) and functional (Ashkenazi et al., 2012; Rosenberg-Lee et al., 2015) abnormalities localized to this region. Aberrant activity in children with MD has also been found in inferior parietal cortex, in particular the supramarginal gyrus (Ashkenazi et al., 2012). Given the role of the intraparietal sulcus and inferior parietal cortex in procedural memory (see the section “The Procedural Deficit Hypothesis of Mathematical Disability”), dysfunction of these regions in MD could lead to procedural memory difficulties, consistent with the PDH.

Other portions of the procedural memory network have also been implicated in MD. Inferior and other frontal abnormalities, including of BA 6 and 44, have been found in children with developmental dyscalculia (Rotzer et al., 2008; Ashkenazi et al., 2012; Rosenberg-Lee et al., 2015). Additionally, abnormal activity during calculation has been observed in the basal ganglia, specifically in the caudate nucleus, both in children with developmental dyscalculia and those with Turner Syndrome, which is also associated with math impairments (Molko et al., 2003). Interestingly, basal ganglia lesions have also been associated with acquired acalculia (Delazer et al., 2004; Roşca, 2009). We are not aware of any abnormalities of cerebellar structures associated with MD.

### Difficulties with Aspects of Math That Depend on Procedural Memory

The PDH of MD posits that MD is explained at least in part by the dysfunction of aspects of math that depend on procedural memory. Although research linking MD to this memory system is still sparse, some evidence suggests that certain aspects of math, including some that seem automatized and are characteristically impaired in MD, depend on this system. As discussed above, procedural memory underlies a wide range of functions, including sequences, rules, and categories, and thus various aspects of math could depend on it.

Several aspects of math learning can be linked to procedural memory, most notably arithmetic (e.g., addition or subtraction). The achievement of arithmetic fluency involves children initially using effortful “procedural” strategies (e.g., counting strategies for addition), but eventually automatizing these processes (Siegler, 1996). Although this is often characterized as a shift from effortful strategies to the retrieval of math facts (e.g., “2 + 3 = 5”), it has alternatively been suggested, consistent with learning in procedural memory, that “procedural” strategies simply become automatized, accounting for observed increases in speed (Baroody, 1983, 1984; Fayol and Thevenot, 2012; Barrouillet and Thevenot, 2013; Prado et al., 2014; Thevenot et al., 2016; Uittenhove et al., 2016). It has been additionally suggested that this proceduralization of arithmetic computations is analogous to the proceduralization of computations in grammar (Baroody, 1983), which in fact have been closely linked to the procedural memory system (Ullman, 2004, 2015, 2016). At the brain level, the circuitry involved in procedural memory (Ullman, 2004, 2016) overlaps considerably with the network subserving arithmetic processing [which includes the intraparietal sulcus, inferior parietal cortex, ventrolateral prefrontal cortex (especially BA 44 for automatized processing; Maruyama et al., 2012; Jeon and Friederici, 2015), and the basal ganglia, as well as the medial temporal lobe and other structures; Menon, 2014], underscoring a possible dependence of arithmetic on the procedural memory system (see the section “Future Directions and Conclusion” for discussion of the medial temporal lobes and declarative memory). Finally, consistent with the predictions of the PDH, children with MD have particular problems with arithmetic, especially with its automatization (Geary, 2004). Although these problems have often been characterized as retrieval deficits (Price and Ansari, 2013), they may also be consistent with difficulties automatizing computations in procedural memory.

Other aspects of math skills that are impaired in children with MD might also involve procedural memory. For example, the count sequence, which eventually becomes highly automatized, is difficult to master for children with MD (Geary, 2004). Similarly, magnitude representation seems to be at least partly implicit and learned, depends on the intraparietal sulcus, and is problematic in MD (Price and Ansari, 2013). Future research seems warranted to examine these and other math skills whose dysfunction in MD may be explained by the PDH – in particular math skills that show behavioral and/or neural signatures of procedural memory, such as being implicit, automatized, or reliant on procedural memory brain structures (Ullman, 2016).

As mentioned above, given the varied functional roles of the brain structures that constitute the procedural memory system, abnormalities of the different structures may result in somewhat different specific deficits, though all could lead to impaired automatization. For example, abnormalities of the caudate nucleus could result in problems with early stages of learning math skills, thus potentially precluding their later automatization, whereas neocortical abnormalities, such as of BA 44, may lead to problems processing automatized routines. We believe that future research should be able to identify which brain abnormalities lead to what types of impairments in automatized math skills in MD.

### Difficulties with Procedural Memory in Other Domains

As discussed above, the posited procedural memory dysfunction in MD likely extends beyond the domain of math. In principle, procedural memory impairments could be found in any domain, with the exact manifestation depending on which portions of procedural memory structures are impacted, and which aspects of procedural memory they support. Thus, like the PDH of SLI (Ullman and Pierpont, 2005), the PDH of MD predicts that procedural memory deficits may be found across a range of tasks. These could include, for example, motor skill learning (e.g., in the rotary pursuit task), sequence learning (e.g., in serial reaction time tasks), probabilistic learning (e.g., in weather prediction tasks), or artificial grammar learning; see Ullman and Pierpont (2005) and Ullman (2016). The exact pattern of procedural memory deficits could reveal the nature of the posited procedural memory impairments in MD. For example, recent evidence suggests that the procedural memory deficits in SLI may particularly affect the acquisition of sequences, perhaps especially their consolidation, consistent with the associated grammatical impairments (Hedenius et al., 2011; Hsu and Bishop, 2014; Lum et al., 2014). We are not aware of any published studies examining procedural learning or consolidation in MD, leaving an important gap for future studies to address. Interestingly, however, MD has been linked to motor skill deficits (Rosenberg, 1989), consistent with procedural memory impairments.

### Difficulties with Non-procedural Functions That Rely on Brain Structures Subserving Procedural Memory

Since the brain structures underlying procedural memory also subserve other, non-procedural, functions, abnormalities of these structures may additionally result in deficits of these functions – with the nature and extent of the deficits depending on which portions of which structures are affected, and what functions they subserve (see the section “The Procedural Deficit Hypothesis of Mathematical Disability”). In the in-depth examination of the PDH of SLI presented by Ullman and Pierpont (2005), a number of such functions were examined. Consistent with extending the PDH to MD, some of these, as well as others, have also been found to be impaired in this disorder, including working memory (Ashkenazi et al., 2013b), attention (Ashkenazi and Henik, 2010; Henik et al., 2011), inhibitory control (Espy et al., 2004), and temporal processing (Vicario et al., 2012). Future research should examine the extent to which non-procedural functions that depend on procedural memory brain structures implicated in MD are affected in the disorder.

### Comorbidity with Other Developmental Disorders That May be Explained by the PDH

The PDH predicts that the posited MD deficits of procedural and non-procedural functions such as of reading, grammar, motor skills, and attention may result in comorbidities between MD and disorders affecting these functions, where these disorders are explained by abnormalities of brain structures underlying procedural memory. Here, we lay out these and related predictions (going beyond the basic claims that partially motivated the PDH of MD), briefly review the literature, and point out gaps in the research.

As discussed above, MD is highly comorbid with dyslexia (Wilson et al., 2015). Nearly two-thirds of children with math difficulties also have reading difficulties (Lewis et al., 1994). Conversely, about one-third of children with reading problems also have math problems (Lewis et al., 1994). Even children with dyslexia with math scores in the normal range show subtle deficits in arithmetic performance (Simmons and Singleton, 2008), and utilize immature strategies for arithmetic problems (Boets and De Smedt, 2010). The PDH predicts common brain abnormalities between MD and dyslexia, and possibly shared etiologies as well. Indeed, like MD, dyslexia is associated with abnormalities of inferior parietal regions (including the intraparietal sulcus), inferior frontal regions (including BA 44 and BA 6), and the basal ganglia (in particular the caudate nucleus) (Eckert et al., 2003, 2005; Richlan, 2012). Further, candidate susceptibility genes for dyslexia (e.g., ROBO1) also appear to contribute to math difficulties (Mascheretti et al., 2014).

Evidence also suggests comorbidity of MD and SLI. Individuals with MD may show indications of SLI (Archibald et al., 2013), while conversely, and better studied, individuals with SLI show various math impairments (Fazio, 1994, 1996, 1999; Arvedson, 2002; Donlan, 2003; Cowan et al., 2005; Donlan et al., 2007). As expected by the PDH, SLI, like MD, is associated with abnormalities of procedural memory brain structures, in particular the basal ganglia (especially the caudate nucleus) and inferior frontal structures (including BA 44 and BA 6), as well as (though more weakly) inferior parietal abnormalities (Ullman and Pierpont, 2005; Ullman et al., accepted). However, to date less research has examined MD comorbidity with SLI than with dyslexia, leaving an important gap for future research.

The PDH also predicts that individuals with dyslexia or SLI should tend to show particular difficulties in aspects of math that depend on procedural memory. Indeed, problems with arithmetic have been found in both dyslexia (Simmons and Singleton, 2008; Boets and De Smedt, 2010) and SLI (Fazio, 1996; Donlan et al., 2007). Additionally, difficulties with the count sequence have been found both in dyslexia (Ackerman et al., 1990; Gobel and Snowling, 2010) and SLI (Fazio, 1994, 1996).

The nature and extent of the comorbidities between MD and either dyslexia or SLI should depend on which procedural memory structures underlie each disorder. For example, if MD is caused primarily by procedural memory dysfunction from parietal abnormalities (see above), whereas SLI is characterized mainly by frontal/basal-ganglia insults (Ullman and Pierpont, 2005; Ullman et al., accepted), the likelihood of their comorbidity will be lower than between disorders with abnormalities in the same procedural memory structures. Interestingly, dyslexia, like MD, is strongly associated with parietal abnormalities (in particular of the left inferior parietal lobe; Richlan, 2012), perhaps helping explain the high comorbidity between these two disorders.

Other disorders may also be expected to be comorbid with MD. In brief, any neurodevelopmental disorder involving abnormalities of brain structures underlying procedural memory could be comorbid with MD, with the likelihood of comorbidity depending to what extent the same (portions of) structures are affected in both disorders. Indeed, at least DCD and ADHD are promising candidates, since both are associated with abnormalities of procedural memory structures (Krain and Castellanos, 2006; Kashiwagi and Tamai, 2013; Peters et al., 2013; Sidlauskaite et al., 2015), and both have been linked to math difficulties (Kaufmann and Nuerk, 2008; Gomez et al., 2015).

## Future Directions and Conclusion

In sum, the PDH provides a set of clear testable predictions for MD. Importantly, our theoretical and empirical understanding of the PDH in language disorders promises to facilitate the investigation of the PDH in MD, even beyond the predictions laid out above. For example, previous work in language disorders suggests that consolidation problems of procedural memory (Hedenius et al., 2011) may also be important in MD. Moreover, the hippocampus-based declarative memory system, which appears to remain relatively spared in SLI and dyslexia (Ullman and Pullman, 2015), may also be important in MD: not only because in language disorders it plays compensatory roles for procedural memory-based impairments (Ullman and Pullman, 2015), but also because it seems to underlie aspects of learning math facts (Cho et al., 2012; Qin et al., 2014). Indeed, such a role for declarative memory is expected, given its importance in learning idiosyncratic information such as facts (Ullman, 2016). More generally, the roles of both declarative and procedural memory in math warrant further investigation, both in MD and TD children, since math, like language, must be largely if not entirely learned, and these are arguably the most important learning and memory systems in the brain (Ullman, 2004, 2016). That is, just as the PDH may be extended from language disorders to MD, the declarative/procedural (DP) model of language (Ullman, 2004, 2016) may be extended to an analogous DP model of math. Finally, research on language disorders suggests that understanding the roles of procedural and declarative memory may lead to important diagnostic and therapeutic advances in MD (Ullman and Pierpont, 2005; Ullman and Pullman, 2015).

Although we emphasize that we are not claiming that all MD is explained by the PDH, we suggest that the hypothesis may offer a substantial amount of explanatory power, and that it provides a useful theoretical framework that may advance our understanding of the disorder.

## Author Contributions

TE and MU contributed equally in conceiving of and writing the manuscript.

## Conflict of Interest Statement

The authors declare that the research was conducted in the absence of any commercial or financial relationships that could be construed as a potential conflict of interest.
